# Medication adherence to oral iron therapy in patients with iron deficiency anemia

**DOI:** 10.12669/pjms.323.9799

**Published:** 2016

**Authors:** Cigdem Gereklioglu, Suheyl Asma, Asli Korur, Ferit Erdogan, Altug Kut

**Affiliations:** 1 Cigdem Gereklioglu, MD. Department of Family Medicine, Baskent University Medical School, Adana, Turkey; 2 Suheyl Asma, Assistant Professor, Department of Family Medicine, Baskent University Medical School, Adana, Turkey; 3 Asli Korur, MD. Department of Family Medicine, Baskent University Medical School, Adana, Turkey; 4 Ferit Erdogan, MD. Department of Family Medicine, Baskent University Medical School, Adana, Turkey; 5 Altug Kut, Associate Professor, Department of Family Medicine, Baskent University Medical School, Ankara, Turkey

**Keywords:** Iron deficiency anemia, Oral iron therapy, Side effect

## Abstract

**Objective:**

This study aimed at investigating the factors affecting medication adherence in patients who use oral iron therapy due to iron deficiency anemia.

**Methods:**

A total of 96 female patients in fertile age with mean age of 30±10.1 years (range 18-53) who were admitted to Family Medicine Clinic between 01 January and 31 March 2015 and who had received iron therapy within the recent three years were enrolled in the study. Data were collected through a questionnaire form.

**Results:**

Of the patients, 39 (40,6%) were detected not to use the medication regularly or during the recommended period. A statistically significant relationship was found between non-adherence to therapy and gastrointestinal side effects and weight gain (p<0.05).

**Conclusion:**

Medication adherence is deficient in patients with iron deficiency anemia. The most important reason for this seems gastrointestinal side effects, in addition to weight gain under treatment.

## INTRODUCTION

Iron deficiency (ID) is evident in more than 1.5 billion people worldwide. It is an important health issue in developing countries like Asia, Africa, and South America and a frequent cause of anemia even in Western Europe.^1^,^2^ In the Western world, while iron deficiency anemia (IDA) is often multi-factorial, menstruation is the commonest single cause. Reduced dietary intake of iron, bleeding from the gastrointestinal tract, mal-absorption (particularly in celiac disease), pregnancy and blood donation are other frequent causes.^3^

IDA is associated with worsened quality of life, impaired physical and cognitive performance.^4^,^5^ Effective treatment of patients with IDA is therefore extremely worthwhile. Oral iron salts are the most readily available way of replacing iron. Taken once or twice a day in tablet form, they are the first line treatment for most indications.^6^ Patients discontinue the use of oral iron therapy because of side effects and lack of long-term adherence to medication in iron deficiency anemia.^7^ The main purpose of this study was to investigate the factors affecting medication adherence in IDA.

## METHODS

This was a single center, cross-sectional study. Patient records were accessed from the page which was formed for patients with IDA for use of authorized personnel only in Data Management System of the Hospital. Record Supervision Committee of Baskent University Research and Training Center controlled data integrity for verification and reliability. This study was approved by Baskent University Institutional Review Board (Project no: KA 15/334) and supported by Baskent University Research Fund.

Standard medication use was defined as using a total of 200 mg ferrous sulfate preparations daily in two equal doses via per-oral route when the patient was fasting. A structured questionnaire method formed to test medication adherence in iron deficiency was used. The questionnaire was composed of questions evaluating whether medications were used regularly and the physician informed the patients about the duration of therapy. The questions about discontinuation of the drug due to relief of symptoms, not being informed about duration of therapy, gastrointestinal side effects (nausea, vomiting, distention, abdominal pain, constipation, diarrhea), weight gain were asked for assessment of treatment-related side effects and the influence of side effects on medication adherence. The questionnaire forms were filled out with face-to-face interviews. All interviews were conducted by the same family physician.

The patients who did not have a definite diagnosis for IDA, who could not exactly remember their medication use, who were diagnosed with a malignant disease, severe gastrointestinal disease, psychiatric disease and who had to use any other medications concurrently were excluded from the study.

### Statistical analysis

SPSS 17.0 package program (SPSS Inc. Chicago, Ilinois, USA) was used for statistical analysis. Categorical variables were summarized as number and percent. Fisher Exact test was used for comparison of categorical variables.

## RESULTS

A total of 96 female patients with mean age of 30±10.1 years (range 18-53) who were admitted to Family Medicine Outpatient Clinic between 01 January 2015 and 31 March 2015, who had received oral iron therapy within the recent three years due to IDA were included in the study. Out of 96 patients, 39 (40.6%) were detected not to use the medication regularly or during the recommended period. Of these patients, 3 (3,1%) stated that they were not informed about the duration of treatment, 4 (4,1%) stated that they discontinued treatment as her symptoms relieved, 36 (37.5%) stated that iron medications caused gastrointestinal side effects and of them, 24 (66,6%) stated that they did not continue medications due to these side effects, 36 (37.5%) patients stated that the medication caused weight gain and of them, 15 (41,6%) stated that they did not comply with the therapy due to this reason (Table I and II). Some patients reported more than one reason for discontinuation of therapy. There were three patients in intersection set of gastrointestinal side effects + weight gain, two patients not being informed about duration of therapy + relieving symptoms, two patients in not being informed about duration of therapy + gastrointestinal side effects. A statistically significant relationship was found between incompliance to therapy and gastrointestinal side effects, weight gain (p<0.05).

**Table-I:** Reasons for discontinuation therapy.

*Reason*	*Number (n):96*	*Percent (%)*
Not being informed about duration of therapy	3	3.1
Relief of symptoms	4	4.1
Weight gain	15	15.6
Gastrointestinal side effects	24	25.0
Gastrointestinal side effects + Weight gain	3	3.1
Not being informed about duration of therapy + Relieving symptoms	2	2.0
Not being informed about duration of therapy + Gastrointestinal side effects	2	2.0

**Table-II:** Distribution of patients according to discontinuation therapy due to side effects.

*Side effect*	*Number of the patients who continue the drug*		*Number of the patients who discontinue the drug*		*p*

	*N:36*	*%*	*N:36*	*%*	
Gastrointestinal side effects	12	33.3	24	66.6	0.0001
Weight gain	21	58.3	15	41.6	0.0001

## DISCUSSION

Iron deficiency anemia is among the most common cause of anemia worldwide, particularly in women. Iron deficiency occurs when iron losses exceed its intestinal absorption. This happens in patients with decreased iron intake, mal-absorption of iron, increased demand for iron or through ongoing iron loss.^8^ Patients with ID are commonly prescribed oral iron preparations because of convenience and low cost. Oral iron salts are the most readily available way of replacing iron. Taken once or twice a day in tablet form, they are the first line treatment for most indications.^9^ The dose of oral iron for IDA should be 30-80mg elemental iron daily, given for 3-6 months, and for longer if the cause of iron deficiency is on-going.^10^ Iron therapy is used to replenish iron stores and restore hemoglobin concentrations to normal, thereby preventing and treating symptoms arising from IDA. While most patients respond well to oral iron preparations, a substantial minority have side effects which make them adhere poorly to their treatment.

Long-term use of oral iron is limited by side effects including nausea, vomiting, constipation, and metallic taste; these side effects are frequent and, although not severe, are often worrisome to patients. Gastro-intestinal symptoms, which occur in up to 30% of people taking oral iron, include nausea, flatulence, abdominal pain, constipation and diarrhea. The clinical impression that these side-effects are dose-related has not been confirmed in a recent meta-analysis.^9^ In our study, 37.1% of our participants were detected to experience gastrointestinal side effects and ratio of discontinuation of therapy due to this reason was found as 21.3%. Although iron preparations are best absorbed when the patient has not eaten, they can be taken after food to reduce gastro-intestinal side-effects. The gastro-intestinal side effects described above should be taken seriously as even mild symptoms may reduce adherence to oral iron supplementation. There is limited evidence to suggest that switching to an alternative oral product can reduce side effects. Despite the lack of supportive meta-analytic data, a dose reduction is sometimes effective and can be equally efficacious in replenishing iron stores.^9^,^11^-^13^ Modified-release preparations of iron are licensed for once-daily dosage, but have no proven therapeutic advantage over conventional formulations. Contrary to some reports, meta-analysis suggests that they are no better tolerated than standard formulations.^9^,^14^ Several new formulations of oral iron are the focus of clinical trials and show promise in relation to efficacy and tolerability but they are not yet routinely available.^15^,^16^

Despite the absence of literature data about iron preparation-related weight gain, patients may have worries about weight gain. In our study, 38.2% of the patients stated that iron therapy lead to weight gain and 12.4% were detected not to comply with therapy for the same reason.

## CONCLUSION

Medication adherence to oral iron therapy was found to be insufficient in IDA, one of the most common cause of anemia in primary care. The leading cause seems gastrointestinal side effects followed by worries about weight gain, inadequate information about duration of therapy. Gastrointestinal side effects may be reduced by dose reduction or taking the pills after meals. The patients who have worries about weight gain may be stated that they could increase their physical activity after hematologic parameters are improved and they may be advised not to eat high calorie food.
